# Quantifying the Severity of Facial Trauma in a Bulgarian Cohort Using the Comprehensive Facial Injury Score

**DOI:** 10.3390/reports9030263

**Published:** 2026-08-10

**Authors:** Ventseslav Ralev

**Affiliations:** Maxillofacial Surgery Unit, MBAL “Dr. Ivan Seliminski”, “Hristo Botev” Str. 1, 8800 Sliven, Bulgaria; ralev@dentist.bg or ralev@maxillofacial.bg; Tel.: +359-888-646-003

**Keywords:** CFI score, duration of the hospital stay, Glasgow Coma Scale, time spent in the operations room, fractures of the facial bones

## Abstract

**Background/Objectives:** The present investigation was designed and conducted with the aim to evaluate the statistical significance of the Comprehensive Facial Injury (CFI) score in relation to total surgical time (ST), length of hospital stay (LOS), and the presence of head injury in patients with fractures of the maxillofacial skeleton. **Methods:** The study includes 332 patients with maxillofacial fractures, a subset of whom also had associated head injuries. The CFI score was calculated for each patient. The Kolmogorov–Smirnov test was applied to assess the distribution of score values within the cohort. The Kruskal–Wallis H test was used to compare ST (minutes), LOS (days), and Glasgow Coma Scale (GCS) scores across different CFI score groups. Statistical significance was defined as *p* < 0.05. **Results:** The majority of the patients were male, at 77.1%, with 22.9% female. The mean age was 36.4 years, with a standard deviation of 19.3 years. Definitive surgical treatment was performed in 58.7% of patients, while the remaining 41.3% were managed conservatively (with closed reduction and external fixation) or received no surgical intervention. A statistically significant association was found between the CFI score and both ST and LOS (*p* < 0.001). Associated head injury was found in 33 patients or 9.9%. Furthermore, significant correlations were observed between CFI score values and Glasgow Coma Scale scores (*p* < 0.001) as well as the presence of head injury (*p* < 0.001). **Conclusions:** In conclusion, the CFI score provides a straightforward and comprehensive framework for assessing both operative duration and hospitalization length in patients with maxillofacial trauma. Moderate correlations between the CFI score and all investigated variables support its reliability in assessing trauma severity. The CFI score may therefore be useful in guiding treatment planning and developing standardized clinical protocols. However, its predictive capacity requires further validation in future studies.

## 1. Introduction

Measurement in medicine and biology is the quantitative assessment of biological characteristics in living organisms. Once measured numerical values are determined, they are categorized and assigned scores within a specific scoring system. Scoring systems aid in estimating the severity of the disease, the prognosis, the risk of complications, the treatment response and the end results. The mathematical calculations based on the scoring systems could lead to evidence or rejection of a scientific hypothesis, and in this way, an evidence-based approach can be established. Maxillofacial injuries, whether isolated or concurrent with other traumas, represent a substantial portion of emergency department and hospital admissions globally. These injuries pose a significant socio-economic burden due to high treatment costs, extended hospital stays, and prolonged periods of missed work. Furthermore, the severity of facial trauma directly dictates surgical planning, operative timing and duration, intensive care unit (ICU) utilization, and the patient’s overall recovery trajectory.

Many researchers from different countries have aimed to create scoring systems that describe the volume of the trauma and ultimately give some direction as to the treatment that should be applied. Canzi et al. [1] proposed a comprehensive facial injury (CFI) score, with the aim to measure the severity of maxillofacial injuries. Building on the study by Canzi et al. [1,2,3], CFI score values were investigated in a group of patients in India by Rawat et al. in 2022 [4]. The present study explores the values of the CFI score among patients with fractures of the maxillofacial skeleton in Bulgaria and the correlation of the index with the length of hospital stay in a high dependency unit and an intensive care unit, surgical time, and Glasgow Comma Scale values.

Both total operating room time and hospitalization duration serve as key performance indicators for trauma team proficiency and healthcare management requirements [5,6,7]. The presumption is that the more severe injuries require a longer surgical time and more days until the patient is released from the hospital, resulting in a longer hospital stay. These two variables are considered as the most appropriate for measurement of the trauma severity. In this way, each new measurement index must have the strongest possible correlation with ST and LHS. There is scientific support for using length of hospital stay and, to a lesser extent, operative time/resource use, as indirect indicators of trauma severity. However, an important nuance from the literature is that these are considered proxy (outcome) indicators, not primary anatomical severity measures like the different scoring systems. The evidence is less direct, but in the trauma literature a longer operative time is generally associated with more complex injuries, a greater surgical difficulty and a higher complication risk.

In many scientific articles, the operative time is used as an indirect severity indicator—especially in maxillofacial trauma studies—though usually alongside length of stay and complication rates. Longer operative time is a predictor of higher complication rates [8]; this is important for clinicians—more important than the description of the trauma in documentation and in scientific articles. Length of hospital stay is widely accepted as an indirect indicator of trauma severity, with longer hospitalization being associated with more severe injuries and complicated clinical courses. Several studies have demonstrated a significant correlation between injury severity scores and hospital stay duration [9]. Especially in polytraumatic cases, longer operative time means that the injury is more severe [10].

The facial skeleton includes multiple bones interdigitating with each other at suture lines. Just one joint is present—the temporomandibular joint. The maxillofacial skeleton is a multifunctional structure. In traumatic events, it functions analogously to a vehicle’s crumple zone. By absorbing and dissipating the kinetic energy delivered by an impacting agent, the facial bones effectively cushion the blow, thereby mitigating severe trauma to the cranium and underlying brain tissue [11,12]. Contrary to the cushioning theory, recent studies indicate that the facial skeleton may funnel impact forces directly into the neurocranium, thereby exacerbating the severity of accompanying traumatic brain injuries [13,14,15,16]. A lower Glasgow Coma Scale (GCS) score serves as a critical indicator for suspected concomitant traumatic brain injury [17].

The objective of this study was to analyze the distribution of CFI scores within a Bulgarian cohort presenting with facial fractures and concomitant injuries, while comparing key clinical performance indicators across the study population.

## 2. Materials and Methods

This study employed a single-center, retrospective, observational design. The cohort included all patients with reported maxillofacial fractures at the emergency department or the Maxillofacial Surgery department of the “Dr. Ivan Seliminski” hospital in the city of Sliven, Bulgaria, which can be considered as a level-one trauma center. All the traumas were treated between May 2011 and March 2026 in the department of Maxillofacial Surgery. Soft tissue injuries were much more frequent than bone fractures, as is generally the case [18,19,20]. However, the biological properties of any facial trauma presenting broken bones are quite different than those of isolated soft tissue; this was the reason for including only patients with bone fractures in the present investigation. The traumatic cases with only isolated soft tissue injuries were excluded. No patient was recalled for an additional examination, except for those who had complaints regarding treatment—even years after it was conducted. The investigator extracted the relevant data into a standardized data sheet. This review was limited strictly to patient medical records, and the primary data sheet was completely de-identified, omitting all personal identifiers such as names and registration numbers.

This study was conducted in accordance with the ethical principles of the Declaration of Helsinki. Due to the retrospective, observational study design using entirely de-identified and anonymized medical records from routine clinical data at the Oral and Maxillofacial Surgery Department, MBAL “Dr. Ivan Seliminski”, specific ethical approval and retrospective informed consent were not required under Bulgarian institutional regulations. No generative artificial intelligence was used in this investigation.

This study evaluated 332 patients with maxillofacial trauma, encompassing isolated bone fractures, combined hard and soft tissue injuries, and cases with or without concomitant head injuries. All patients were admitted to the “Dr. Ivan Seliminski” Hospital during the study period. Surgical interventions were performed by a single surgeon and categorized into definitive approaches—such as open reduction and internal fixation (ORIF) or complex laceration suturing—and conservative management, including closed reduction, intermaxillary fixation, splinting, zygomaticomaxillary fracture reduction, or uncomplicated suturing. Selected clinical cases required no intervention. Associated head injuries were managed concurrently by the respective neurosurgical or other medical specialists.

The primary outcomes evaluated in relation to the CFI score were total surgical time (ST) and length of hospital stay (LHS) within the high-dependency unit (HDU) or intensive care unit (ICU). Additionally, the associations between facial trauma, the Glasgow Coma Scale (GCS) score, and concomitant head injuries were analyzed.

**Figure 1:**
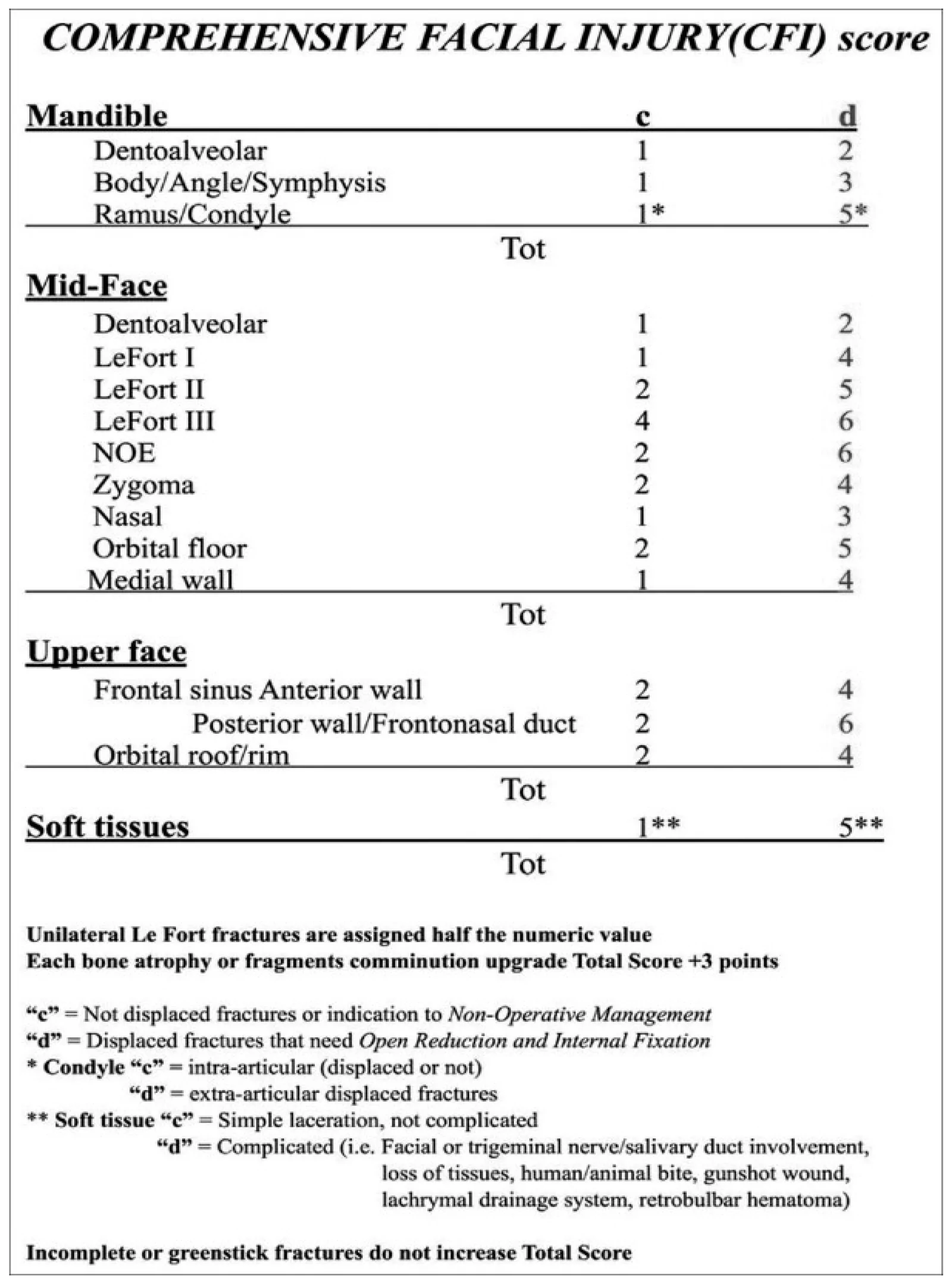
The CFI scale comprises a structured checklist that provides a comprehensive anatomical and functional classification of maxillofacial injuries. The asterisk symbol on the ramus and condyle value means that 1 point is assigned when the fracture is intra-articular (displaced or not) and 5 points—when the fracture is displaced and needs an open reduction and internal fixation. Two asterisks about the soft tissues mean that the wound is complicated—affects the facial or trigeminal nerve branches, or the salivary ducts, there is a tissue defect, it is a bit or gunshot wound or there is a retrobulbar hematoma or lacrimal drainage system injury. The present table was published for the first time in Canzi et al. in 2019 [1] and has been used in other clinical studies [2,3,4] for evaluating facial trauma severity. The figure was taken from Canzi et al. [1] according to the Creative Commons Attribution-Noncommercial-Share Alike 4.0 License. The score can be calculated for cases with isolated soft tissue injuries, but these were not included in the present study.

The values of the CFI score were calculated for each patient after evaluating every case record file and computer tomography scan image stored in the system of the department of Image Diagnostics. This required summing of the evaluated score for each clinical case—see Figure 1.

The whole cohort was divided into six clusters according to the calculated CFI scores:

- Cluster 1—score ≤ 5;
- Cluster 2—score between 6 and 10;
- Cluster 3—score between 11 and 15;
- Cluster 4—score between 16 and 20;
- Cluster 5—score between 21 and 25;
- Cluster 6—score over 25.

Data normality was evaluated using the Kolmogorov–Smirnov test. Because the Comprehensive Facial Injury (CFI) scores and other continuous variables demonstrated a non-Gaussian distribution, non-parametric methods were applied for all subsequent comparisons. Continuous variables were expressed as either mean ± standard deviation or median with interquartile range (Q1–Q3), while categorical data were presented as frequencies and percentages. The Kruskal–Wallis H-test was employed to compare surgical time (ST, minutes), length of hospital stay (LHS, days), and Glasgow Coma Scale (GCS) scores across the six distinct patient clusters. Statistical significance for all CFI score categories was established at a two-tailed *p* value equal to or less than 0.05. All statistical analyses were performed using the Statistical Package for the Social Sciences, version 31.0 (SPSS Inc., an IBM Company, Chicago, IL, USA). Microsoft Access and Excel programs (Office 2024, Microsoft Inc.—Vermont, CA, USA) were used for primary database construction and for some additional calculations.

## 3. Results

**Figure 2:**
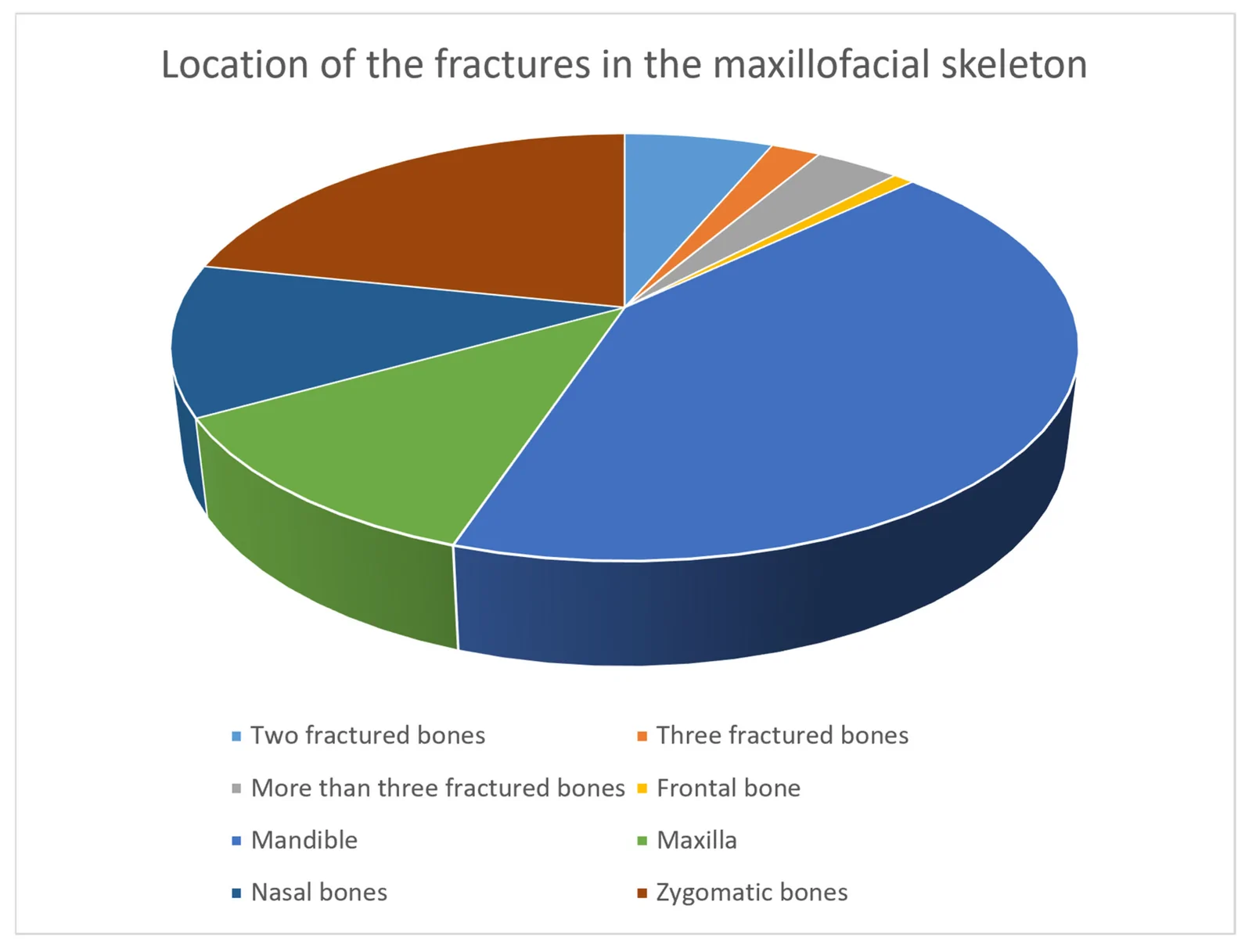
Diagram of the different locations of the fractures in the maxillofacial skeleton.

**Figure 3:**
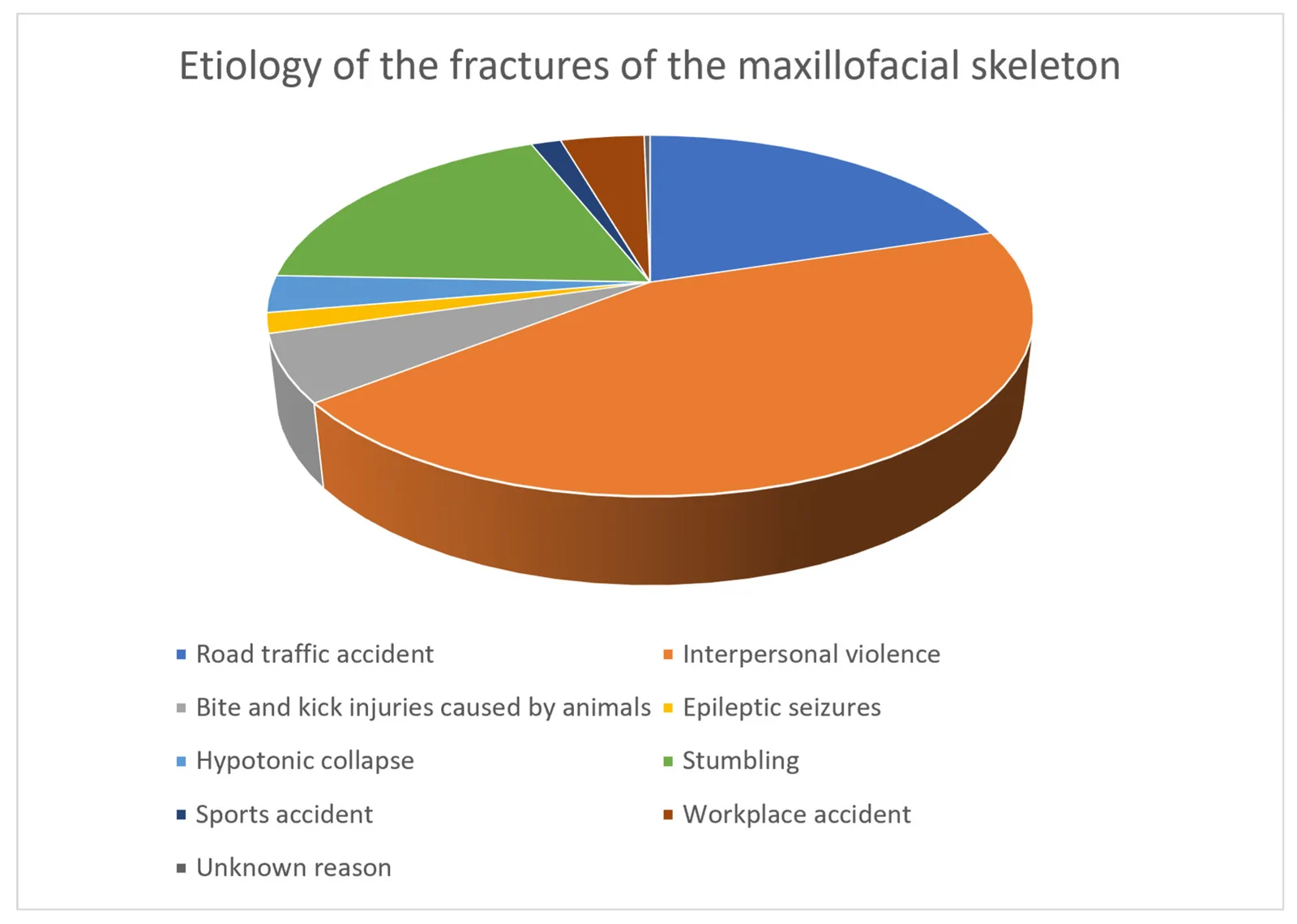
Etiology of the fractures of the present cohort.

**Figure 4:**
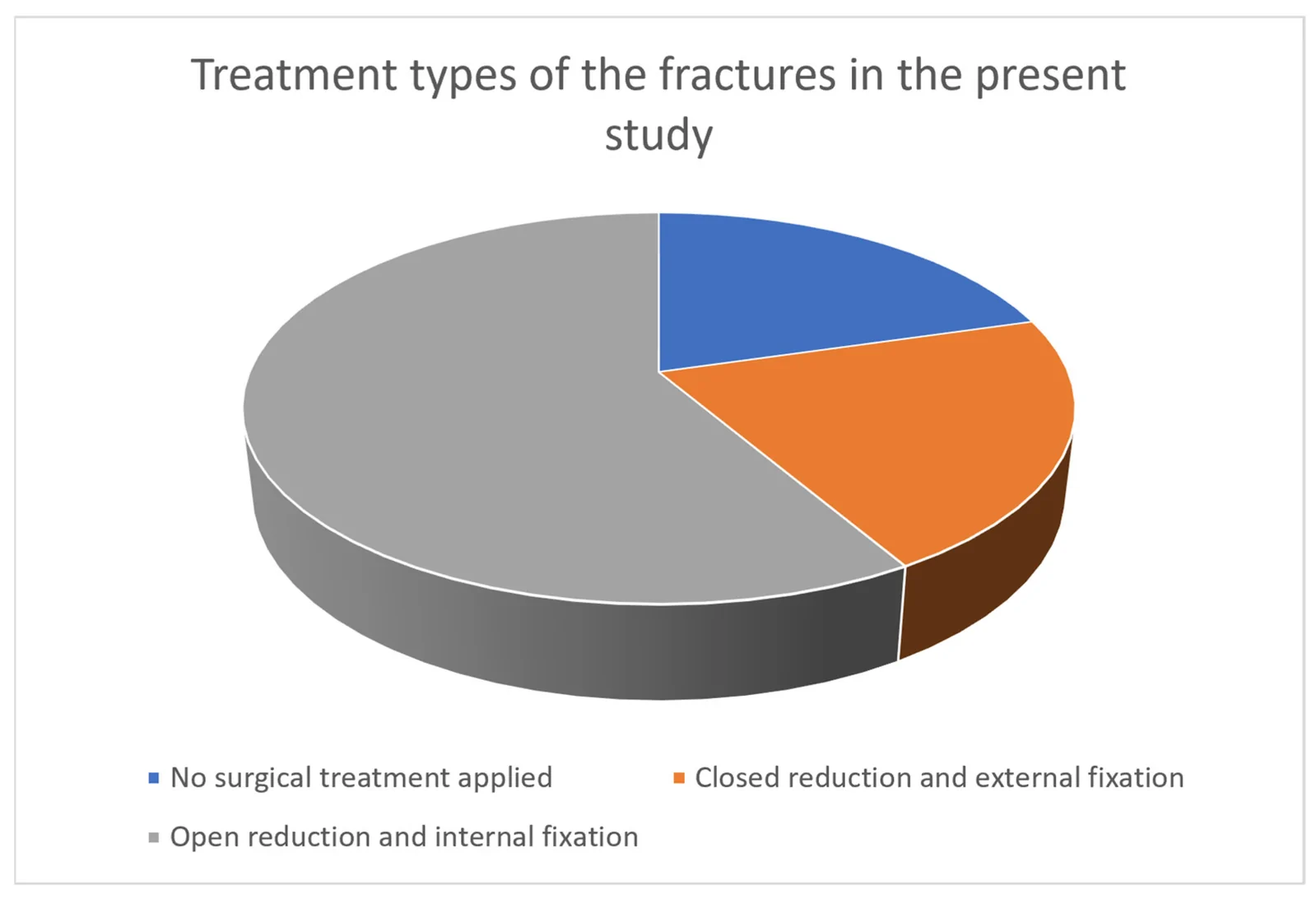
Treatment of the fractures included in the study.

The study cohort included 332 patients, 256 males (77.1%) and 76 females (22.9%), of all ages. The mean age was 36.4 years, with a range between 1 and 88 years and a standard deviation of ±19.3 years. Table 1 and Figure 2 present the location of the fractures in the present study. Forty patients or 12% were found to have more than one fractured bone in the whole maxillofacial skeleton. Table 2 and Figure 3 present the etiology of the fractures from the present investigations. One fracture was caused by a dog’s bite—on a 9-year-old child—with a soft tissue injury of the nose and the forehead combined with an opened fracture of the nasal bones. The other 19 fractures from the animals’ group were caused by horse kicking or biting. One patient was found by the police on the street unconscious with a fractured mandible—with an unknown reason of the injury. Table 3 and Figure 4 present three different kinds of treatment were applied to the clinical cases.

**Figure 5:**
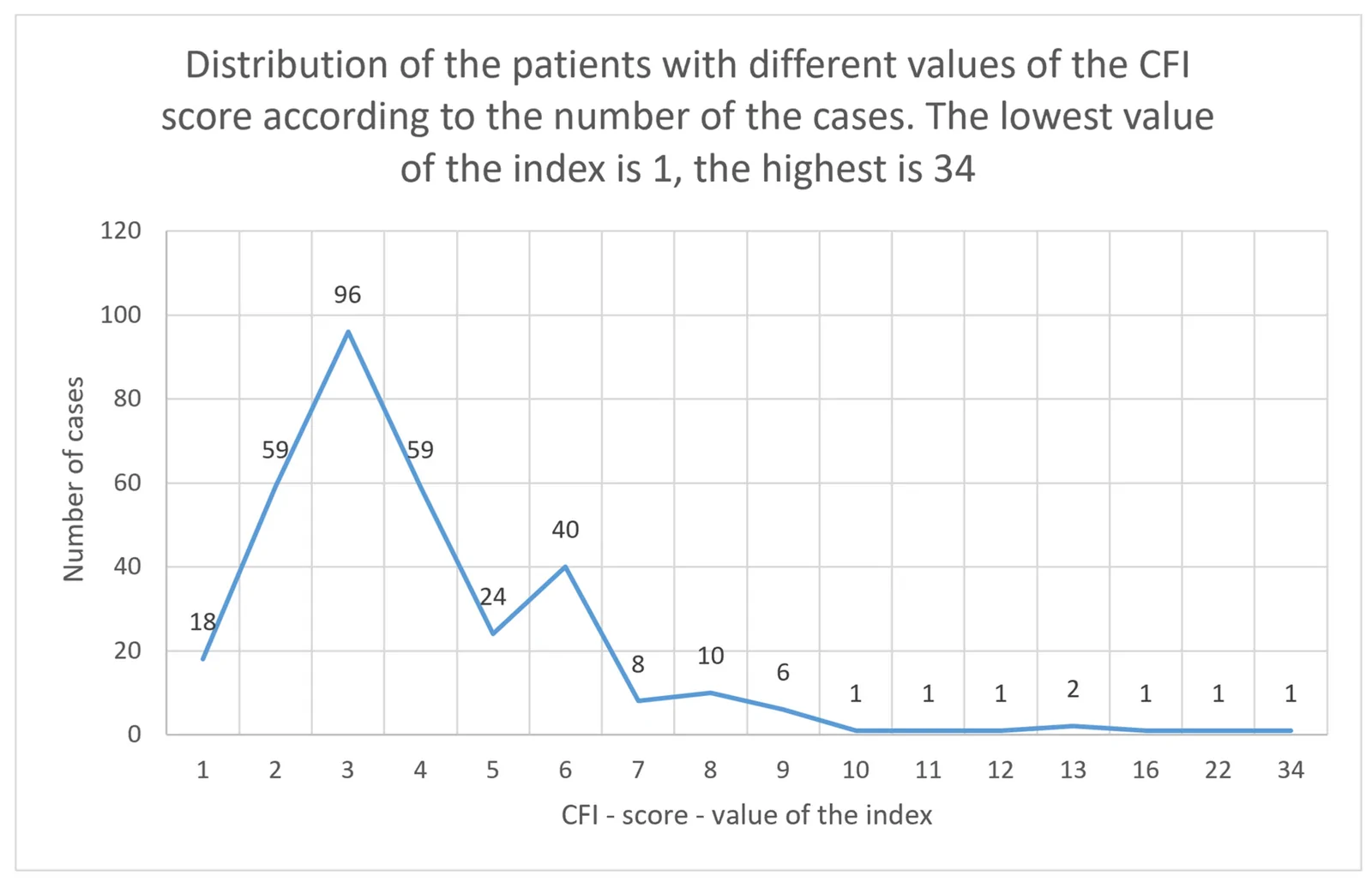
Distribution of the different values of the index according to the number of the patients.

The Kolmogorov–Smirnov test was utilized to evaluate data distribution, revealing a non-Gaussian pattern—*p* < 0.001. There is a heavy tail on the right side due to some clinical cases with high CFI score values; the score could not be less than one, and in this way, there is nothing to exclude from the lower values. But if the tail in the high levels of the score is excluded and the highest value is limited to five, the distributions become a classical Gaussian one. The highest number of cases have a CFI score of three—this is because this value is applied to a single fracture line of the mandible, and the lower jaw is the most common fractured bone of the viscerocranium [21]. A simple visual analysis (Figure 5) shows that the distribution curve looks like a left transferred typical Gaussian distribution. The mean CFI score of all cases included in the database was 4.1 ± 2.9. The median of the whole database was three, and the mode value was also three, due to the high percentage of the injuries to the mandible including just one fracture line, with no soft tissues lacerations.

The following outcomes were studied across Section 3.1, Section 3.2, Section 3.3 and Section 3.4.

### 3.1. The Time Spent in the Operating Room

**Figure 6:**
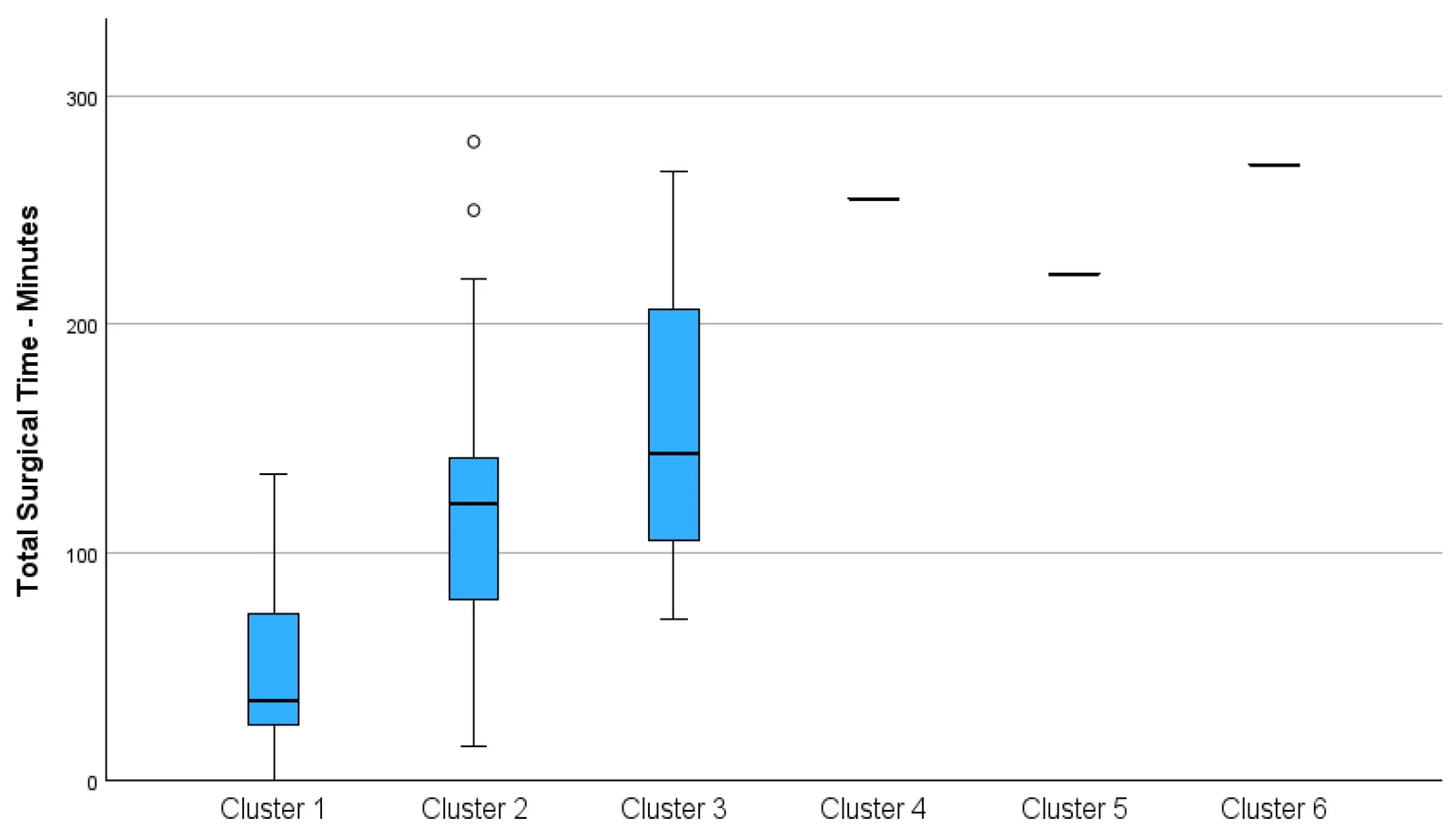
Distribution of total surgical time values among the six clusters, presented as box plots.

**Figure 7:**
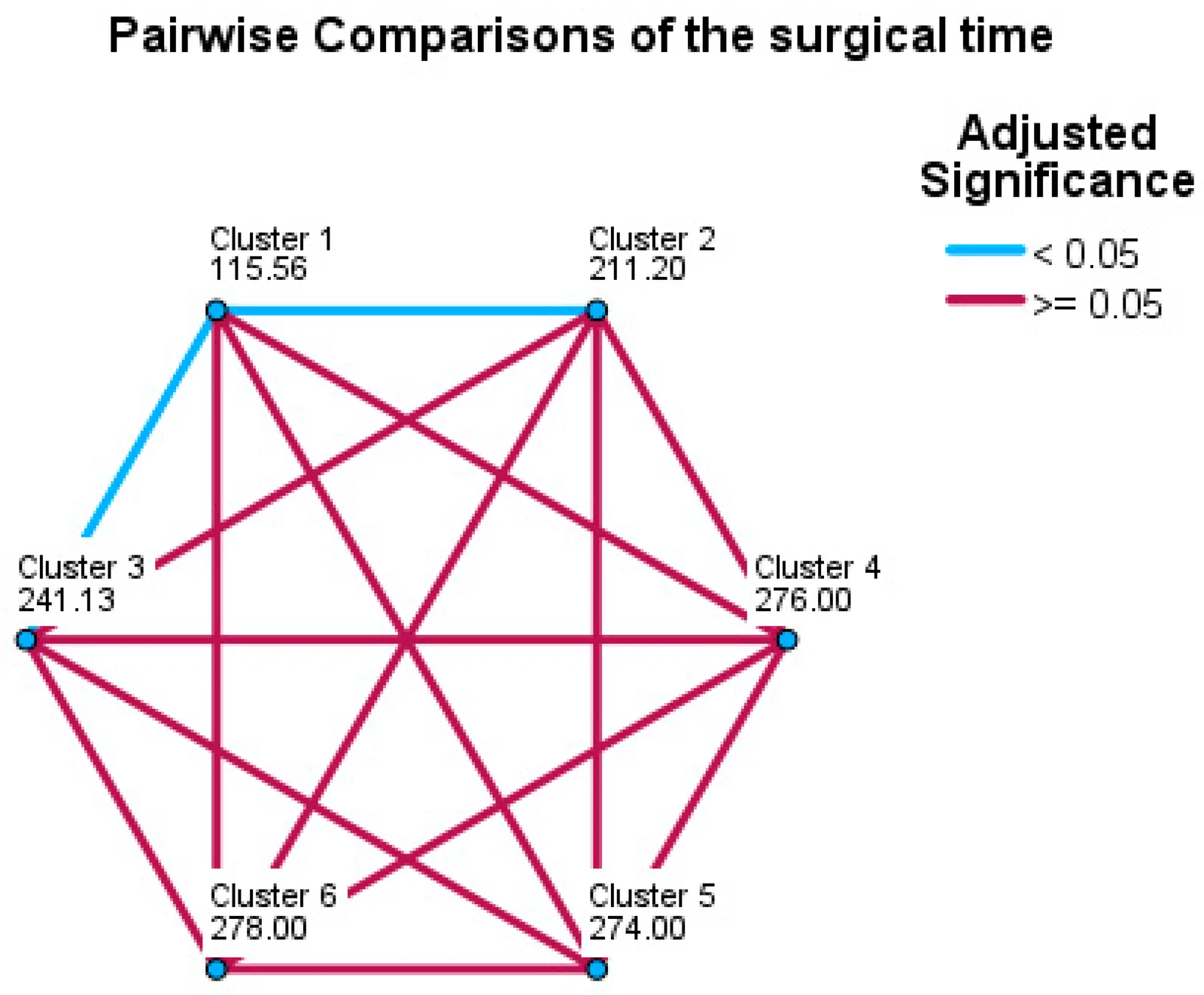
Pairwise comparisons of the mean surgical time, as determined by the Kruskal–Wallis test. Each node shows the sample average rank of the surgical time.

**Figure 8:**
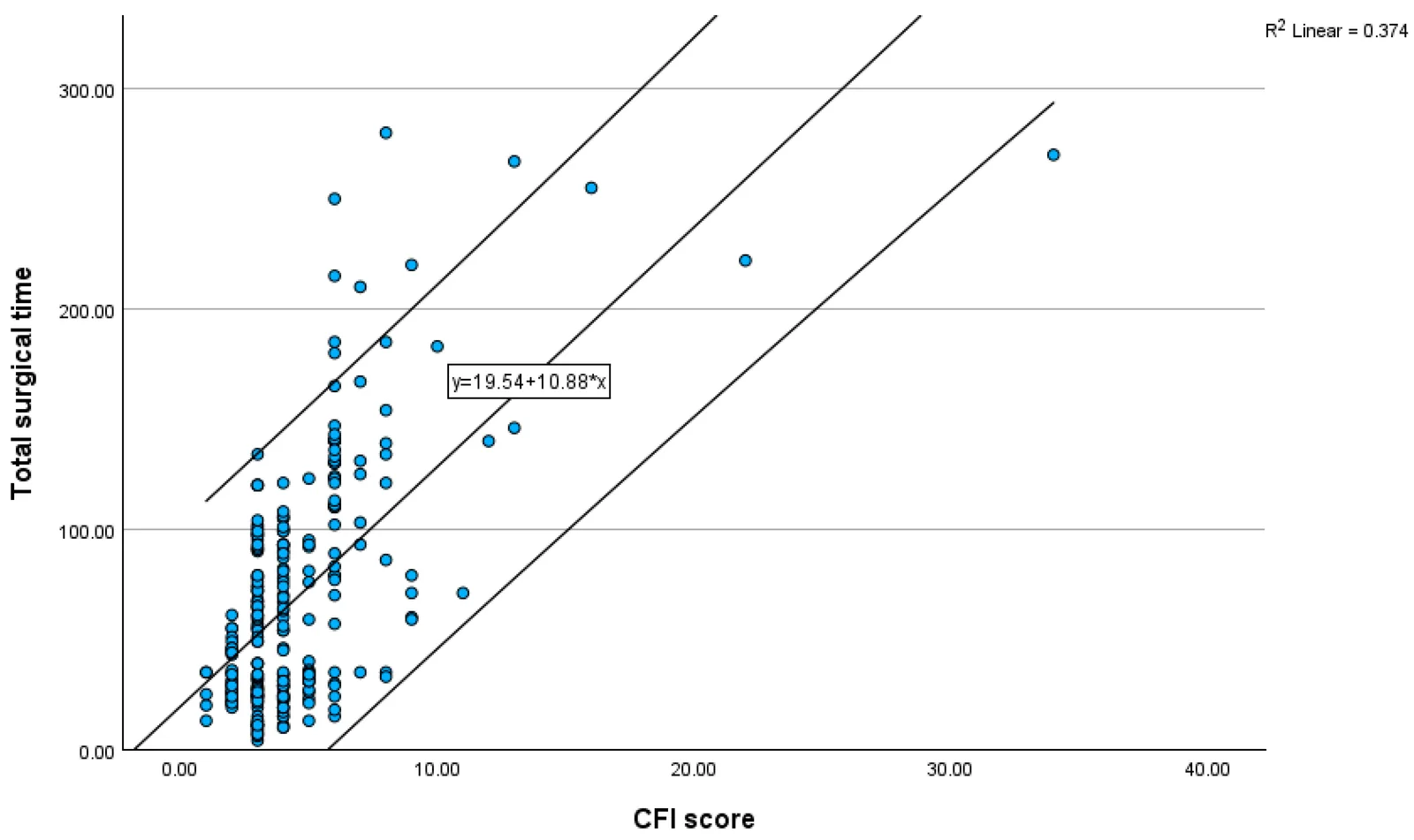
Linear regression analysis demonstrated a proportional increase in surgical time relative to advancing CFI values, *p* < 0.001.

The box plot analysis (Figure 6) clearly demonstrates the observed outcomes—an increase of the median values of the surgical time when the CFI score values increased to 20 points. After that can be seen a pronounced conservation of the interquartile distance amplitude—obviously, longer surgical time is not needed when the trauma is more severe than one level. Figure 7 presents the pairwise comparisons of the mean surgical time, as determined by the Kruskal–Wallis test. The differences are between clusters 1 and 2 and clusters 1 and 3. No other statistically significant differences were found—*p* > 0.05. Figure 8 illustrates a simple linear regression model involving 332 patients, with the CFI score and total ST (minutes) designated as the independent and dependent variables, respectively.

The median value grows until cluster 4; after that, it remains nearly constant. This means that even the most severe facial fractures do not need long operations, in contrast to surgery in other areas of the human body. No microvascular reconstruction was conducted in any clinical case—a type of surgery that requires a long time in the operating room. This type of facial trauma treatment is rarely needed and is only required due to severe tissue defects, obtained mainly in war time or as a result of natural disasters.

The Kruskal–Wallis H-test revealed a statistically significant difference in total ST across the six CFI score clusters (*p* < 0.001); see Table 4. Further pair-wise comparison (shown in Table 5) indicated that the ≤5 CFI score is statistically significantly different from the 6 to 10 score (Cluster 2, where *p* < 0.05 between the two groups) and 11–15 score (Cluster 3, and *p* < 0.05) in terms of duration of surgery. It is noteworthy that the pairwise comparison between all other clusters did not show any difference at *p* > 0.05. This means that the ST grows until level one and then does not increase. The coefficient of linear regression R^2^ has a value of 0.374; in medical research, this is considered as a good model to predict the values of the dependent variable. The longest operation was on a case from Cluster 2 and had a surgical time of 280 min. The shortest one was on a case from Cluster 1 and took 4 min.

### 3.2. Duration of Hospitalization in the High-Dependency Unit

**Figure 9:**
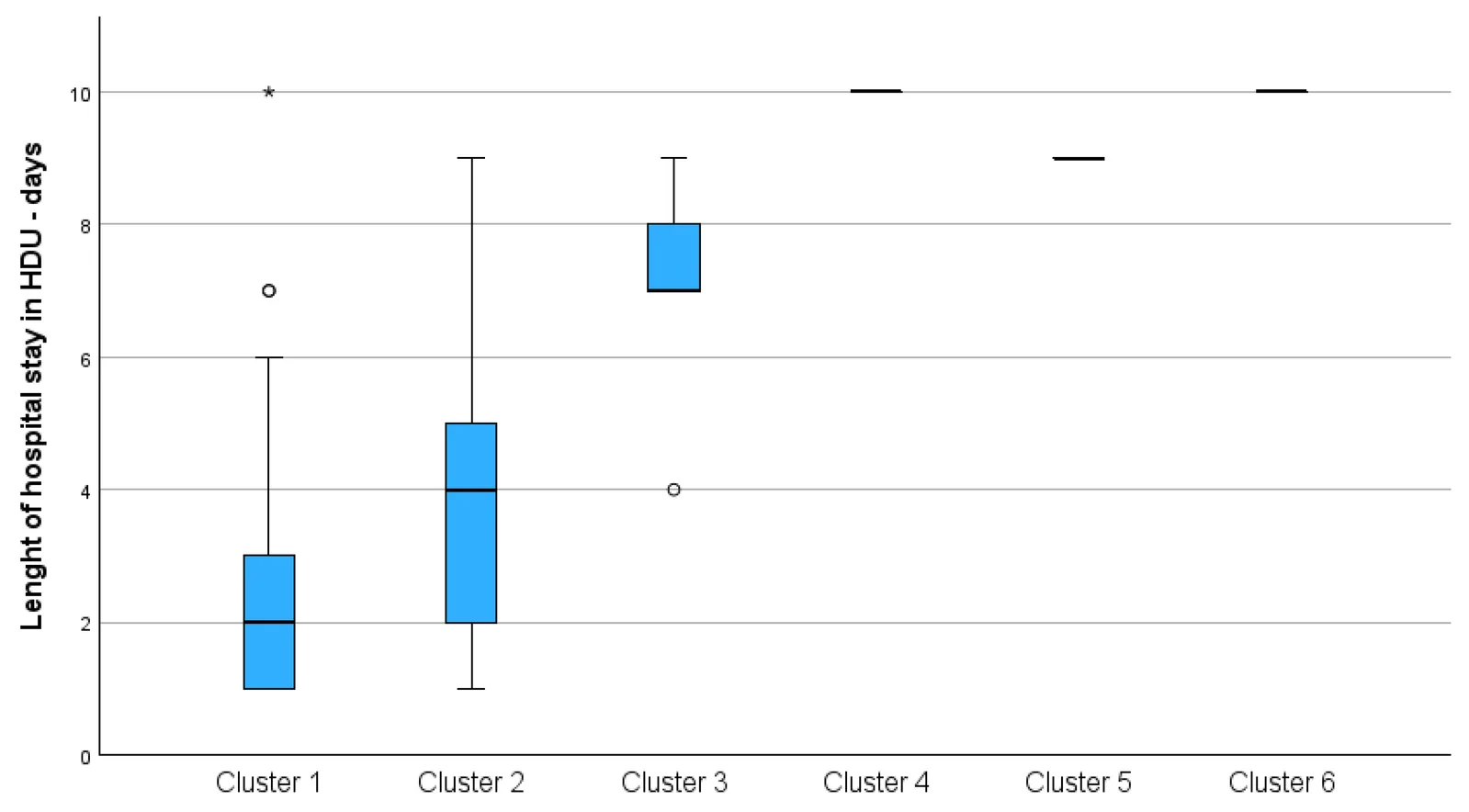
Distribution of hospitalization duration among the six patient clusters, presented as box plots. The distribution between the clusters is the same as that related to the surgical time—no patient had to stay in the hospital for an extended period.

**Figure 10:**
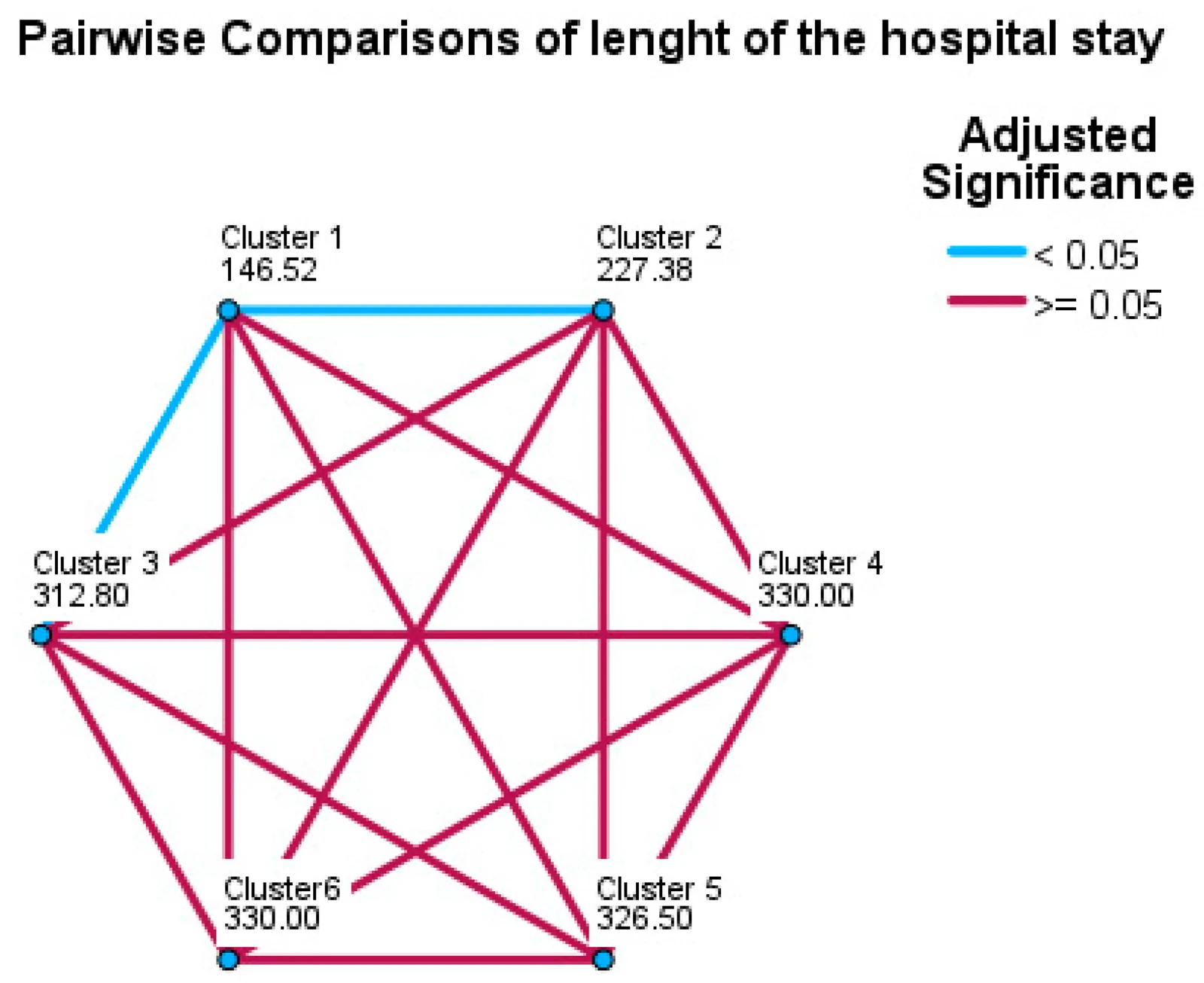
Pairwise comparisons of the mean values of the length of the hospital stay. The differences are between clusters 1 and 2 and clusters 1 and 3. No other statistically significant differences were found—*p* > 0.05. Each node shows the sample average rank of the length of the hospital stay.

**Figure 11:**
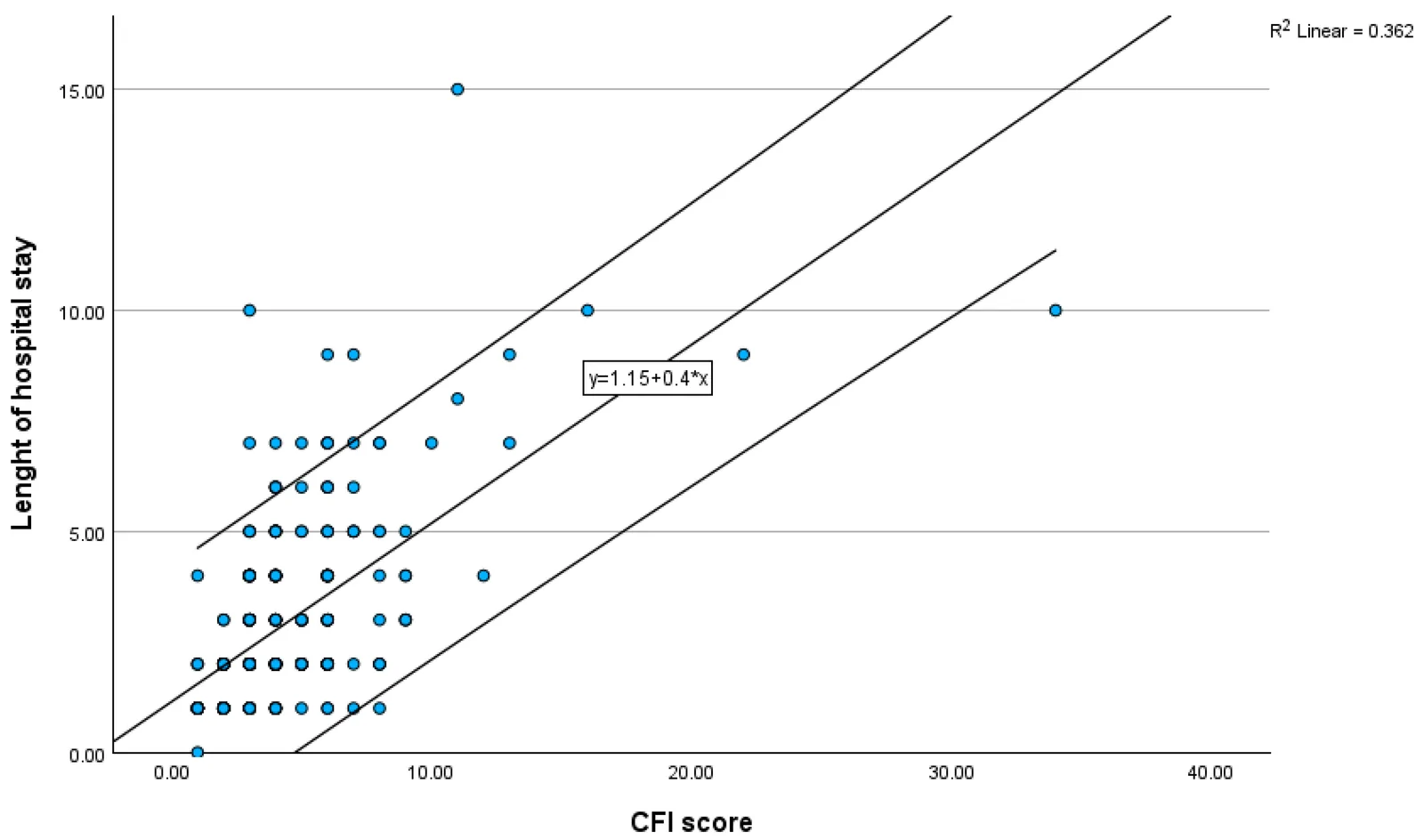
Linear regression of the length of the hospital stays. When the CFI score increases, the length of the hospital stay also increases—*p* < 0.001.

The box plot highlights a progressive upward trend in HDU length of stay (HDU-LOS) corresponding with advancing CFI cluster numbers, peaking at a CFI score of 25; see Figure 9. The length of hospital stay (LHS) also differed statistically significantly across the clusters (*p* < 0.001) (Table 6). On pair-wise comparison, it was found that the ≤5 CFI score is statistically significantly different from the 6 to 10 score (Cluster 2—*p* < 0.05) and 11–15 score (Cluster 3—*p* < 0.05). Between all the other clusters there was not a statistically significant difference—*p* > 0.05 (Figure 10). This distribution and tendency are similar to those concerning the surgical time—increasing until level one and then remaining constant. The existence of a positive linear regression between these two variables is confirmed; see Figure 11. The coefficient of linear regression R^2^ has a value of 0.362—again, characteristic of a good model for prediction of the values of the dependent variable.

### 3.3. Intensive Care Unit Length of Stay

The data were classified based on those with facial injuries and those with both facial and head injuries among the CFI score clusters, but subgroup analysis could not be carried further due to a lack of samples in each group. Only 15 (4.5%) patients from the whole group had to stay in the ICU. The Kruskal–Wallis test showed a high statistically significant difference between the six clusters (*p* < 0.001)—every patient from clusters 4, 5 and 6 had to stay in the ICU for several days. Just 1.15% of the patients in cluster 1 needed an ICU stay. It is interesting that there was no statistically significant difference between the length of the stay in the ICU, at *p* = 0.185, a value above the level of 0.05.

### 3.4. Association with Glasgow Coma Scale (GCS) Scores and Concomitant Head Trauma

**Figure 12:**
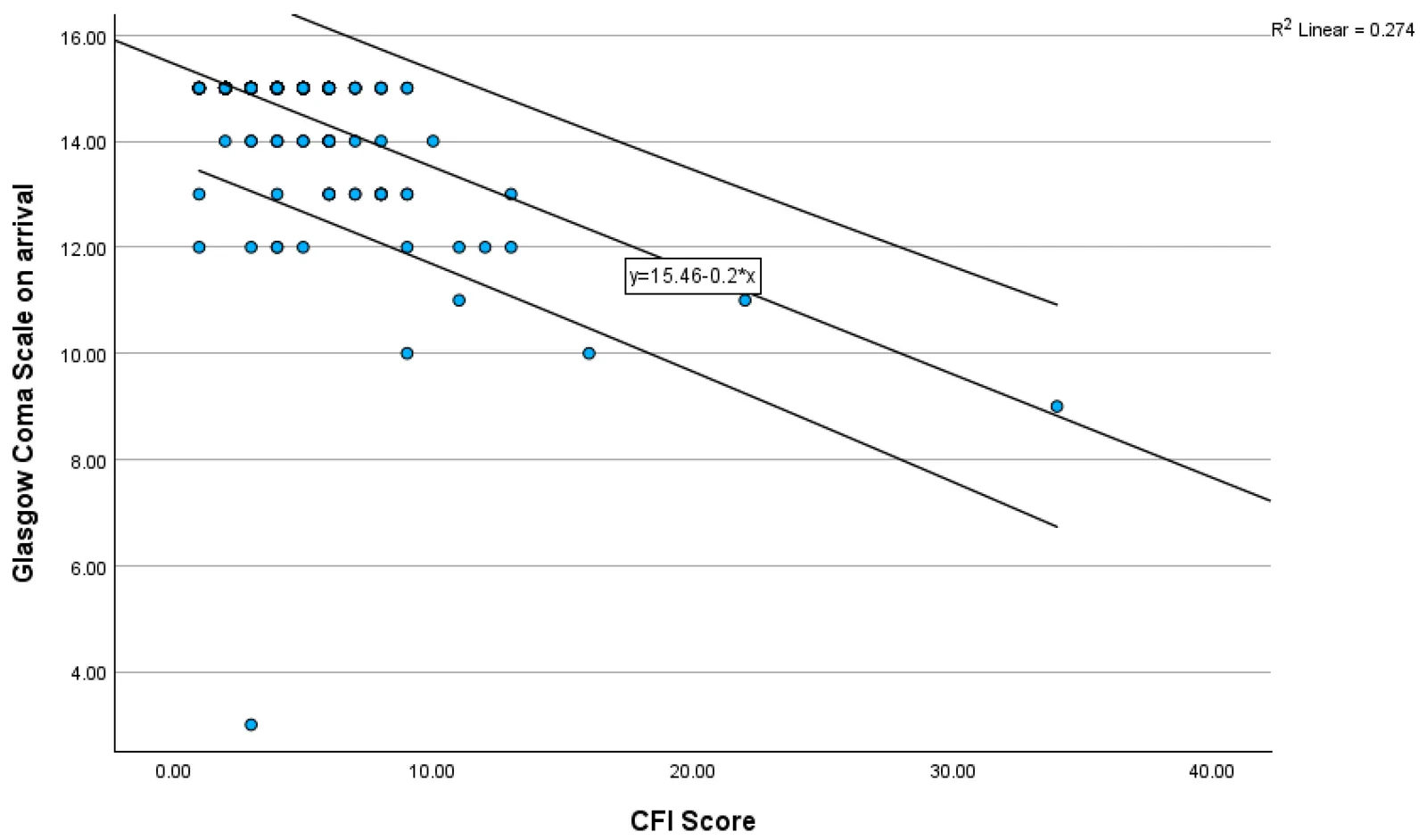
Linear regression analysis between the CFI score and admission GCS; patients presenting with a higher CFI index demonstrated a corresponding decrease in GCS scores.

Baseline GCS scores at admission also differed statistically significantly across the six clusters—*p* < 0.001. CFI scores 6–10 (*p* = 0.020) and 11–15 (*p* = 0.017) were statistically significantly different from the up-to-5 CFI score group. Linear regression analysis confirmed that a higher CFI score was a significant predictor of a depressed GCS score; see Figure 12. Cases showing a decrease in GCS score (<15) increased from Cluster 1 (3.25%) to Cluster 3 (80%). A total of 33 patients (9.9% of all cases) had an associated traumatic head injury, and that percentage within the six clusters increased with decreasing CFI score—a negative correlation. The association was significant (*p* < 0.001); see Table 4. The coefficient of linear regression R^2^ had a value of 0.274—characteristic of a weak to moderate model of prediction of the values of the dependent variable.

## 4. Discussion

The five existing facial injury severity systems are as follows:

1. Cooter and David Score—described for the first time in 1989 [22].
2. Maxillofacial injury severity score (MFISS)—Zhang, 2006 [23], modified by Zhaohui et al. in 2008 [24], with the idea of being included and operated in easier computer databases.
3. Facial injury severity scale (FISS)—Bagheri, 2006 [25].
4. Revised FISS index—discussed in Zhaohui et al., 2008 [24].
5. Facial fracture severity score—Catapano, 2010 [26].

One additional scoring system could be found in the article of Shetty et al., 2007 [27]—but it describes only the mandible fractures and not those of all fractures of the maxillofacial skeleton. All these systems had some characteristic drawbacks. Which one of them is the best and describes the severity of the trauma in the most punctual way—that question is the subject of future studies including more patients with different types of injuries.

To date, the CFI score is the only system that evaluates facial fractures in a different way in cases where an open reduction and internal fixation is needed and where a displaced fracture is not present; conservative methods or no intervention at all would provide a better treatment result. This is the main reason for choosing the CFI score for the present study among all other trauma evaluation systems found in the scientific literature. There are many guidelines and scientific articles that provide guidance to surgeons on when to operate and when conservative treatment should be applied—Li et al., 2019 [28]; Schneider et al. [29]; Rohit et al., 2021 [30]; Ikeda et al., 2021 [31]. However, the evaluation of every clinical case remains subjective. The main principle is that more severe injuries more often require an open reduction and internal fixation; if this could be addressed by the scoring system early on, the trauma severity could be better described by the appropriate scale. The CFI score gives much higher values, especially to fractures of the mandibular condyle and ramus, where an open reduction and internal fixation is needed, and notes where more conservative treatment would be better—values of appropriately 5 and 1 [1].

The overall surgical time and the length of hospital stay both increase when the CFI score increases up to 15 points, as does the relative share of the patients who must stay in the intensive care unit; in clusters 4, 5 and 6, every patient stayed for several days in the ICU. This positive outcome helps validate the importance of the CFI scale in expressing the severity of facial injury and the requirements for hospital resources.

For the most common cases, simple scoring systems (and simple methodology in general) work better; however, the anatomy of the facial cranium is not a simple case, and a scoring system cannot not be simplified endlessly without losing some important information.

Length of hospital stay (LHS) is a critical outcome metric that reflects the economic burden imposed on patients, the consumption of in-patient hospital resources, and overall institutional turnover. In assessing predictive models, Aita et al. [5] evaluated the Facial Injury Severity Scale (FISS) relative to LHS, concluding that the index is somewhat oversimplified and exhibits restricted predictive utility except in cases with higher severity scores.

Although the Maxillofacial Injury Severity Score (MFISS) is a validated metric for correlating treatment costs and hospitalization length, its universal utility is constrained. As noted by Zhang et al. [23] and Zhaohui et al. [24], variations across socioeconomic landscapes and healthcare systems complicate cross-study comparisons and reproducibility. Conversely, Nishimoto et al. [32] and Shetty et al. [27] identified a positive correlation between MFISS scores, total operative duration, and length of hospital stay, emphasizing its specific utility in evaluating mandibular trauma severity.

Traditionally, the facial skeleton was conceptualized as a protective barrier designed to absorb kinetic energy and shield the neurocranium from severe trauma, a theory supported by Foolmar et al. [33], Lim et al. [34], and Mulligan et al. [35]. However, the contemporary literature disputes this paradigm. Investigative work by Keenan et al., Kraus et al. [13], and Martin et al. [14] suggests that facial structures—specifically the midfacial and upper facial regions—may instead serve as conduits that transmit impact forces directly to the neurocranium, thereby elevating the risk of concomitant brain injury.

The hypothetical protective role of the facial skeleton against intracranial trauma has long been a subject of debate. To address this question, Keenan et al. [13] evaluated a cohort of 3388 bicyclists to analyze the relationship between facial fractures and traumatic brain injuries (TBIs). Their findings provided no empirical evidence that facial fractures shield the brain; conversely, the data demonstrated that facial fractures serve as clinical markers for an elevated risk of concurrent neurotrauma.

Within this framework, elevated Comprehensive Facial Injury (CFI) scores likely reflect exposure to greater kinetic energy during the traumatic event, which culminates in more severe maxillofacial trauma. Concurrently, this heightened energy transference propagates to intracranial structures, potentially precipitating concomitant neurological or head injuries. The number of patients with concomitant traumatic brain injuries is small—just 33 patients; thus, in the present investigation, this represents a hypothesis that requires a larger sample for future research to be confirmed or rejected. Such a mechanism could explain the lower values of the Glasgow Coma Scale observed among patients with higher CFI scores in the present study. However, the value of the R^2^ regression coefficient shows a weak to moderate part of the variation explained by the regression model.

Woriax et al. [36] observed that severe midface fractures correlate with a lower incidence of concomitant intracranial hemorrhages, spinal fractures, pneumothorax, and abdominopelvic trauma. This phenomenon is frequently attributed to the “deceleration effect,” a mechanism wherein the midfacial skeleton acts as an energy-dissipating shield, attenuating the transmission of kinetic energy to the neurocranium, cervical spine, and torso.

Conversely, Joshi et al. [37] demonstrated that the risk of neurological trauma escalates significantly in tandem with declining Glasgow Coma Scale (GCS) scores and an increasing burden of facial fractures. Their data underscore a multifaceted relationship between maxillofacial trauma and intracranial injury, indicating that both fracture severity and spatial distribution are critical determinants of outcomes. Furthermore, the investigators emphasized that future research must prioritize elucidating force transmission mechanisms, identifying specific risk factors for mild traumatic brain injury, and evaluating long-term functional recovery.

The reported incidence of concomitant traumatic brain injury (TBI) among patients with facial fractures spans a remarkably broad spectrum, ranging from 5.4% to 87%; refer to Joshi et al., 2018 [37]. In this clinical context, a low Glasgow Coma Scale (GCS) score serves as a pivotal diagnostic indicator of underlying intracranial trauma. Such injuries remain a primary driver of global mortality and long-term morbidity, imposing a profound socioeconomic burden on healthcare infrastructure.

In the current study, this specific outcome was analyzed within a relatively limited patient subgroup; nonetheless, a statistically significant association was established (Table 4). A progressive reduction in GCS scores was noted alongside escalating CFI values up to a threshold of 15, revealing a clear negative linear correlation (Figure 12). Moreover, the prevalence of depressed GCS scores (<15) within each clinical cluster rose proportionally with increasing CFI scores up to this point. Similarly, the incidence of concomitant head injuries demonstrated a progressive upward trend across higher CFI score clusters up to a value of 15 (Table 4).

Every patient in the ICU stayed a few days (between 1 and 5), until the common health status was stabilized. No patient remained there for a longer period of 10 or 15 days; if the sample was bigger, such kinds of clinical cases would exist. There is no statistically significant difference between the subgroups according to the mean duration of the ICU stay. This is likely due to the common characteristics of the trauma pathology—traumatic patients are generally in a good health, and an acute accident is the primary cause of the disease. In this way, even where severe trauma occurs, the ICU is not necessary—in 2 or 3 days, the patient can be stabilized and referred to the HDU for further treatment [38,39,40]. This situation is different from other pathological processes like those related to oncology and inflammation—the patient comes to the hospital in a damaged status, worsened by the disease over a period of weeks or months, and therefore needs more time in the ICU [41].

### Limitations

The primary limitation of this study lies in the relatively modest sample size and its disproportionate distribution across the distinct CFI score clusters. Additionally, the low incidence of patients with concomitant traumatic brain injuries or those requiring ICU admission may attenuate the statistical power and external validity of these specific sub-analyses. Lalloo et al. in 2020 [42] estimated the incidence of the fractures of facial bones at about 98 per 100,000 people yearly globally. Bulgaria has a population of 6.5 million; thus, yearly, there should be 6370 fractures of facial bones at the national level. Over a period of approximately 15 years, the incidence of facial fractures in the country should be about 95,500 cases. So, the present study includes less than 0.3% of all cases in Bulgaria for the explored period.

All the patients included in the present investigation are treated in the Maxillofacial surgery department in the hospital at Sliven from May 2011 until February 2026—almost 15 years. This is quite a long period and, especially with respect to the surgical time, some impunctualities could appear—the learning curve is not taken into consideration. During this long period, the surgical skills of every operator improved significantly, and the time of the operation (ST) could significantly decrease. In some specific cases, it could increase, because with the growth of clinical experience, the operator sees that sometimes more precision is needed, which requires more time in the operating room. This is an important issue for future research and should be taken into consideration. Some variations could also appear in the length of the hospital stay; the increase in clinical experience in some cases could result in earlier patient release from the hospital, with others treated for a longer time to avoid complications. The hospital admission protocols and the shifting perioperative antibiotic regimens in Bulgaria did not change significantly during the period of the investigation; thus, these factors could not impact the LHS. The only factor that changes is the learning curve of the surgical skills of the operator; this should be the subject of future investigations for longer periods and including a larger number of patients. In many cases, surgical skills could lessen due to aging, vision problems and many other factors; thus, this is also an important theme of future studies.

The whole cohort was divided into six clusters, although the clusters 4, 5 and 6 contain just one clinical case. Cluster imbalance is the central statistical weakness of the present research. However, other cohorts show a similar number of cases [1,4] distributed across the six clusters. This was the reason for accepting the same type of distribution, although clusters 4, 5 and 6 could be merged. The dividing of the cohorts into six clusters, applied for the first time by Canzi [1], could be accepted as a research standard for the future and could be used for a comparison with different cohorts of patients from different countries around the globe. Future studies with a larger number of cases could estimate with greater statistical significance whether this type of cluster division is the best for maxillofacial fracture epidemiology or whether another type with a different number of clusters should be applied. In the present study, the clinical cases with extremely large values for the CFI score a presented more as descriptive case notes than inferential strata.

The small sample size of TBI patients prevents stratified regression to confirm CFI as an independent TBI predictor.

## 5. Conclusions

The results obtained demonstrate an acceptable level of statistical significance, validating that this relatively new scoring system can moderately predict key parameters related to the management of maxillofacial fractures. These include total surgical time and hospital resource utilization, particularly in terms of length of hospital stay, for surgical and conservative treatment approaches.

The model yields diverse clinical applications, ranging from the formulation of targeted therapeutic strategies to the optimization of surgical timing, procedural sequencing, and the prognosis of overall patient outcomes.

The Comprehensive Facial Injury (CFI) score offers a robust anatomical classification of maxillofacial trauma that correlates directly with the modality of treatment administered. Consequently, it represents a valuable instrument for communicating patient morbidity and establishing standardized therapeutic protocols for populations with equivalent injury severity. Furthermore, the CFI scoring system balances ease of implementation with thorough descriptive capacity, rendering it highly viable for widespread adoption in acute trauma centers. This framework optimizes cross-disciplinary communication and promotes standardized clinical decision-making paths.

The formulation of a comprehensive yet intuitive scoring system for grading facial trauma severity deepens the understanding of how associated and concomitant injuries interact. Furthermore, it establishes a framework for future investigations into the systemic and localized effects of simultaneous, multi-regional trauma.

While an elevated CFI score represents a potential risk factor for concurrent head injuries, its predictive utility requires further validation in larger-scale studies to establish a statistically robust association. Furthermore, variability across distinct fracture phenotypes significantly influences the applicability and sensitivity of the scoring system, thereby ensuring it aligns more closely with real-world clinical conditions. This variation is largely driven by divergent biological healing processes, heterogeneous institutional management strategies and, crucially, disparities in treatment-associated expenditures.

The interplay between severity scoring metrics, chosen therapeutic modalities, and definitive clinical outcomes remains a fundamental consideration in systemic trauma evaluation. Within the specific domain of maxillofacial surgery, further investigation is warranted to elucidate the precise strength and nature of this association. Prospective research may identify reproducible clinical patterns and establish refined guidelines to enhance prognostic accuracy. Specifically, these insights will optimize clinical decision-making by clarifying whether operative intervention or conservative management delivers superior outcomes on an individualized patient basis. Ultimately, such advancements would significantly enrich residency training programs and elevate the global standard of trauma care.

## Figures and Tables

**Table 1:** Location of the fractures in the present study.

Location of the Fracture	Number of Patients	Relative Share
Mandible	140	42.2%
Zygoma	72	21.7%
Maxilla	39	11.7%
Nasal bones	38	11.4%
Frontal bone	3	0.9%
Combination of two fractured bones	21	6.3%
Combination of three fractured bones	7	2.1%
Combination of more than three fractured bones	12	3.6%
Common	332	100%

**Table 2:** Etiology of the fractures from the present investigations.

Location of the Fracture	Number of Patients	Relative Share
Interpersonal violence	147	44.3%
Road traffic accident	67	20.2%
Stumbling	61	18.4%
Bite and kick injuries caused by animals	20	6%
Workplace accident	14	4.2%
Hypotonic collapse	11	3.3%
Epileptic seizures	6	1.8%
Sports accident	5	1.5%
Unknown reason	1	0.3%
Sum	332	100%

**Table 3:** Three different kinds of treatment were applied to the clinical cases.

Treatment Types Applied	Number of Patients	Relative Share
Open reduction and internal fixation	194	58.4%
Closed reduction and external fixation	78	23.6%
No surgical treatment applied	60	18%
Sum	332	100%

**Table 4:** The distribution of the studied variables among 6 clusters of CFI scores. Abbreviations: *p*—probability value, *N*—number of patients; ST—Surgical Time; HDU—High Dependency Unit; ICU—Intensive Care Unit; LHS—Length of Hospital Stay; GCS—Glasgow Comma Scale. Row 4 describes the number and the percentage of patients from each cluster needed to be treated for some days in an intensive care unit, with a minimum of one day.

Outcome	Cluster 1 *N* = 259	Cluster 2 *N* = 65	Cluster 3 *N* = 5	Cluster 4 *N* = 1	Cluster 5 *N* = 1	Cluster 6 *N* = 1	*p*
Mean surgical time in minutes	49 ± 31	116 ± 57	156 ± 81	255	222	270	<0.001
Mean LHS in HDU (days)	2.3 ± 1.4	4 ± 2	7 ± 2	10	9	10	<0.001
Mean LHS in ICU (days)	3 (2.66)	4 (1.75)	5 (1.2)	1 (1)	1 (1)	1 (3)	0.185
LHS in ICU %	1.15%	6.15%	100%	-	-	-	<0.001
Mean points of GCS on arrival	14.8 ± 0.9	14.3 ± 1	12 ± 0.6	10	11	9	<0.001
Concomitant head injury	9 (3.5%)	16 (24.6%)	4 (80%)	1	1	1	<0.001

**Table 5:** Values of the different statistical indicators of the mean values of surgical time in the six clusters. Each row tests the null hypothesis that the Sample 1 and Sample 2 distributions are the same. Asymptotic significances (2-sided tests) are displayed. The significance level is 0.050. ^a^: Significance values have been adjusted using the Bonferroni correction for multiple tests.

Sample 1–Sample 2	Test Statistic	Standard Error	Standard Test Statistic	Significance Level	Adjusted Significance ^a^
Cluster 1–Cluster 2	−95,641	11,728	−8155	<0.001	0.000
Cluster 1–Cluster 3	−125,561	40,718	−3084	0.002	0.031
Cluster 1–Cluster 5	−158,436	80,865	−1959	0.050	0.751
Cluster 1–Cluster 4	−160,436	80,865	−1984	0.047	0.709
Cluster 1–Cluster 6	−162,436	80,865	−2009	0.045	0.668
Cluster 2–Cluster 3	−29,920	41,639	−0.719	0.472	1000
Cluster 2–Cluster 5	−62,795	81,333	−0.772	0.440	1000
Cluster 2–Cluster 4	−64,795	81,333	−0.797	0.426	1000
Cluster 2–Cluster 6	−66,795	81,333	−0.821	0.412	1000
Cluster 3–Cluster 5	−32,875	90,197	−0.364	0.715	1000
Cluster 3–Cluster 4	−34,875	90,197	−0.387	0.699	1000
Cluster 3–Cluster 6	−36,875	90,197	−0.409	0.683	1000
Cluster 5–Cluster 4	2000	114,091	0.018	0.986	1000
Cluster 5–Cluster 6	−4000	114,091	−0.035	0.972	1000
Cluster 4–Cluster 6	−2000	114,091	−0.018	0.986	1000

**Table 6:** Values of the different statistical indicators of the mean length of the hospital stay in the six clusters. Each row tests the null hypothesis that the Sample 1 and Sample 2 distributions are the same. Asymptotic significances (2-sided tests) are displayed. The significance level is 0.050. ^a^: Significance values have been adjusted using the Bonferroni correction for multiple tests.

Sample 1–Sample 2	Test Statistic	Standard Error	Standard Test Statistic	Significance	Adjusted Significance ^a^
Cluster 1–Cluster 2	−83,186	12,816	−6491	<0.001	0.000
Cluster 1–Cluster 3	−164,525	41,712	−3944	<0.001	0.001
Cluster 1–Cluster 5	−181,425	92,562	−1960	0.050	0.750
Cluster 1–Cluster 4	−184,925	92,562	−1998	0.046	0.686
Cluster 1–Cluster 6	−184,925	92,562	−1998	0.046	0.686
Cluster 2–Cluster 3	−81,338	42,875	−1897	0.058	0.867
Cluster 2–Cluster 5	−98,238	93,091	−1055	0.291	1000
Cluster 2–Cluster 4	−101,738	93,091	−1093	0.274	1000
Cluster 2–Cluster 6	−101,738	93,091	−1093	0.274	1000
Cluster 3–Cluster 5	−16,900	101,201	−0.167	0.867	1000
Cluster 3–Cluster 4	−20,400	101,201	−0.202	0.840	1000
Cluster 3–Cluster 6	−20,400	101,201	−0.202	0.840	1000
Cluster 5–Cluster 4	3500	130,650	0.027	0.979	1000
Cluster 5–Cluster 6	−3500	130,650	−0.027	0.979	1000
Cluster 4–Cluster 6	0.000	130,650	0.000	1000	1000

## Data Availability

The original contributions presented in this study are included in the article. Further inquiries can be directed to the corresponding author.

## Abbreviations

The following abbreviations are used in this manuscript:

CFI	Comprehensive Facial Injury
ST	Surgical Time
LHS	Length of Hospital Stay
GCS	Glasgow Comma Scale
HDU	High Dependency Unit
ICU	Intensive Care Unit
TBI	Traumatic Brain Injury
